# Unveiling the Sugary Secrets of *Plasmodium* Parasites

**DOI:** 10.3389/fmicb.2021.712538

**Published:** 2021-07-16

**Authors:** Felix Goerdeler, Peter H. Seeberger, Oren Moscovitz

**Affiliations:** ^1^Department of Biomolecular Systems, Max Planck Institute of Colloids and Interfaces, Potsdam, Germany; ^2^Institute of Chemistry and Biochemistry, Freie Universität Berlin, Berlin, Germany

**Keywords:** *Plasmodium*, glycans, glycobiology, glycosylphosphatidylinositol (GPI), O-glycans, N-glycans, malaria, glycocalyx, glycosaminoglycans, host defense

## Abstract

*Plasmodium* parasites cause malaria disease, one of the leading global health burdens for humanity, infecting hundreds of millions of people each year. Different glycans on the parasite and the host cell surface play significant roles in both malaria pathogenesis and host defense mechanisms. So far, only small, truncated *N-* and *O*-glycans have been identified in *Plasmodium* species. In contrast, complex glycosylphosphatidylinositol (GPI) glycolipids are highly abundant on the parasite’s cell membrane and are essential for its survival. Moreover, the parasites express lectins that bind and exploit the host cell surface glycans for different aspects of the parasite life cycle, such as adherence, invasion, and evasion of the host immune system. In parallel, the host cell glycocalyx and lectin expression serve as the first line of defense against *Plasmodium* parasites and directly dictate susceptibility to Plasmodium infection. This review provides an overview of the glycobiology involved in *Plasmodium*-host interactions and its contribution to malaria pathogenesis. Recent findings are presented and evaluated in the context of potential therapeutic exploitation.

## Introduction

Malaria, the disease caused by the parasite *Plasmodium*, kills approximately 400,000 people each year, the majority of which are children under the age of five (World Health Organization [WHO], 2020). While the number of cases declined for many years, a result of effective prevention and therapy, recent years have witnessed a surge in case numbers due to increasing drug resistance of the parasite and rapid population growth in the most severely affected countries (World Health Organization [WHO], 2020). *Plasmodium* species generally display high specificity for their respective host and among the five *Plasmodium* species that infect humans, *Plasmodium falciparum* and *Plasmodium vivax* are by far the most common and lethal for humans (World Health Organization [WHO], 2020). Other *Plasmodium* species infest non-human hosts, including primates, rodents, birds and even reptiles with varying degrees of host specificity. For instance, *Plasmodium knowlesi* infects both macaques and humans, showing an unusual degree of host promiscuity, while *Plasmodium reichenowi* only infects chimpanzees. In mice, *Plasmodium berghei, Plasmodium yoelii*, and *Plasmodium chabaudi* are the most relevant species that have been extensively used as model organisms in malaria research (De Niz and Heussler, 2018).

*Plasmodium* parasites have a complex life cycle involving transmission by an insect vector to the human host where parasites at the so-called sporozoite stage first infect the liver and then develop into the blood stages of the parasite (Maier et al., 2019). Inside the red blood cells (RBCs), parasites mature from ring to trophozoite to schizont stage. At this stage, the RBC ruptures, and merozoites emerge to start the next round of RBC infection. A small number of parasites leave the cycle to form gametocytes that are taken up by the insect vector during a blood meal. Inside the vector, sexual reproduction of the parasite takes place, and ookinetes develop into oocysts, while traversing the mosquito midgut. Upon oocyst rupture, sporozoites are released again and invade the mosquito salivary glands to infect the next host (Maier et al., 2019).

Glycans denote the carbohydrate part of a glycoprotein or glycolipid and consist of monosaccharides linked via glycosidic bonds (Varki et al., 2017). At various life stages of the parasite, glycans present on the surface of parasites and host cells engage in host-parasite interactions (summarized in Table 1). Glycosylphosphatidylinositol (GPI) glycolipids, produced by the parasite in large quantities, contribute to severe anemia and hyperinflammation in the host (Boutlis et al., 2005; Nebl et al., 2005). *Plasmodium* also expresses small, truncated *N/O-*glycosylations on its surface proteins (Bushkin et al., 2010; Kupferschmid et al., 2017). However, the function of GPIs and *N/O-*glycosylations for the parasite is still being debated. At the host surface, sialic acid-containing *N/O-*glycans and glycosaminoglycans (GAGs) studied in detail because they are used by *Plasmodium* as docking sites for invasion and cytoadherence (Orlandi et al., 1992; Pancake et al., 1992; Frevert et al., 1993; Rogerson et al., 1995). In addition, the host glycocalyx and several glycan-binding host proteins, so-called lectins, play a critical role in host defense and malaria susceptibility (Barragan et al., 2000; Hempel et al., 2014; Introini et al., 2018).

**TABLE 1:**
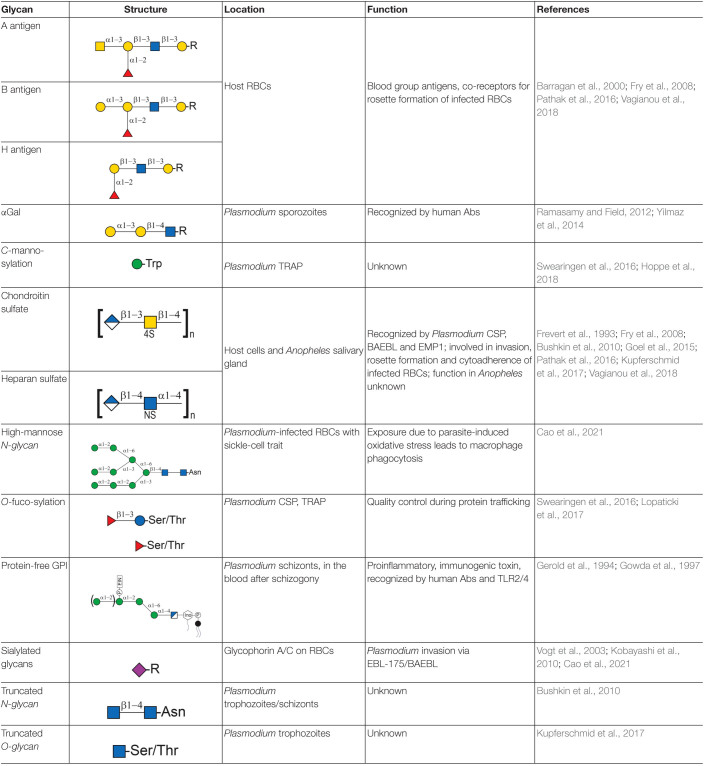
List of all glycans with a role in *Plasmodium* infection.

*For each glycan, the table contains the structure, location, function and references. In case of heterogeneous glycans (e.g., GAGs), example structures are depicted.*

The complexity of glycan–protein interactions at the *Plasmodium*-host interface is further increased by the large number of different *Plasmodium* and host species and most previous work examed the glycobiology of a few selected *Plasmodium* species such as *P. falciparum* or *P. berghei*, further hampering general conclusions. In this article, we mostly focus on *Plasmodium* species that infest humans and rodents. We review recent publications on the glycobiology aspects of the *Plasmodium* life cycle with the aim to disentangle the glycan interactions at the *Plasmodium*-host interface and to point out possible intervention sites for malaria therapy.

## *Plasmodium* Glycosylations

### Biosynthesis and Metabolism

**FIGURE 1:**
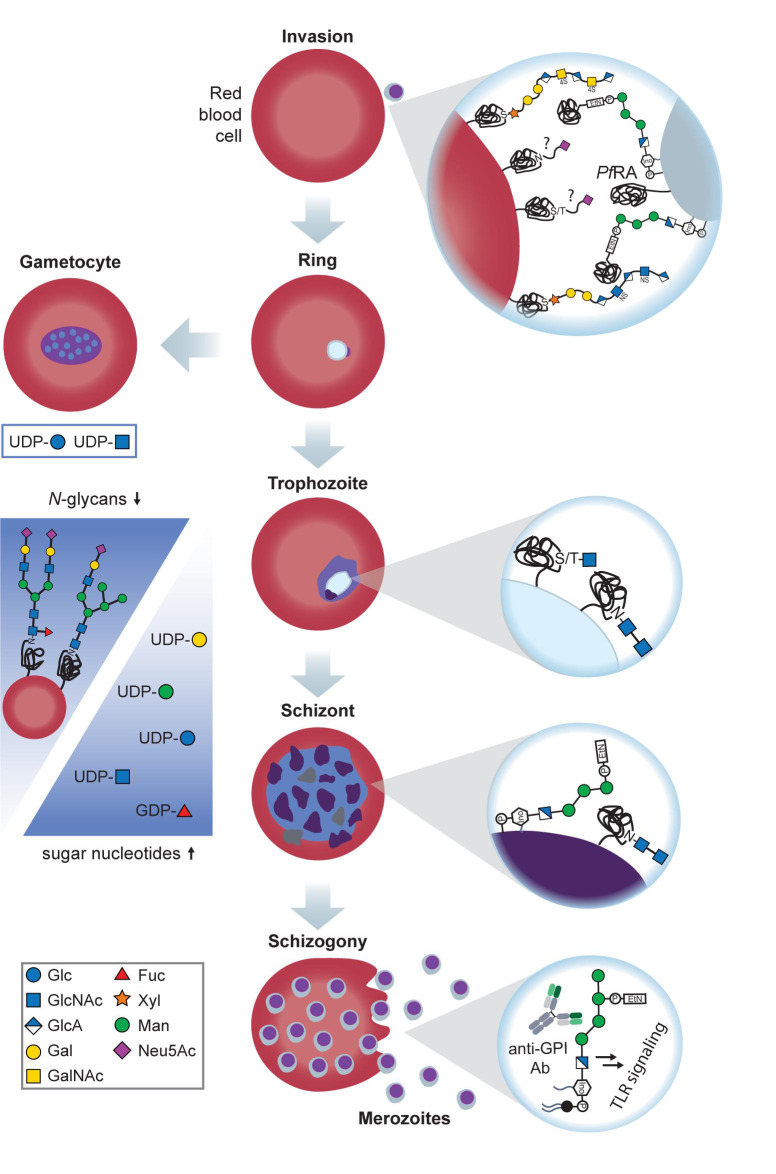
Glycans at the sexual and asexual blood stages of *Plasmodium.* Merozoites recognize RBC GAGs via *Pf*EMP1 and *Pf*BAEBL, an essential interaction for invasion. In addition to GAGs, *Plasmodium* binds to sialylated glycophorin A and C on the RBC surface in the sialic acid-dependent pathway of invasion. Sialic acids are recognized by *Pf*BAEBL and *Pf*EBL-175 on the merozoite surface. After invasion, *Plasmodium* rings either proceed to the trophozoite stage or the gametocyte stage for sexual reproduction (left). Possible gametocyte glycosylation remains to be investigated, but it is known that the levels of UDP-Glc and UDP-GlcNAc are significantly increased at this stage, suggesting a high demand for these building blocks at the sexual life stages. Trophozoite surface glycoproteins possess truncated *O-* and *N-*glycans with 1–2 GlcNAc moieties of unknown function. When progressing to the schizont stage, *Plasmodium* maintains these short *N-*glycans and heavily expresses GPIs, with and without anchored proteins. In addition, *N-*glycans on the infected RBC’s surface decline, and sugar nucleotide levels rise (blue arrows), suggesting that *Plasmodium* increasingly utilizes scavenging pathways to build up the required glycosylations. Upon rupture of the schizont, protein-free GPIs are released together with the merozoites. These GPIs are recognized by macrophages and induce a proinflammatory, toxic response, thereby contributing to anemia.

The common precursors for glycan biosynthesis are sugar nucleotides from which monosaccharides are transferred to nascent glycan chains, proteins or lipids by glycosyltransferases (Varki et al., 2017). Sugar nucleotides generally consist of a nucleotide, such as UDP, GDP, CMP, or CDP connected to a monosaccharide. Metabolic labeling and liquid-chromatography mass-spectrometry helped to identify pools of UDP-GlcNAc, UDP-Glc, UDP-Gal, GDP-Man and GDP-Fuc in *Plasmodium*. The size of these pools increased from ring to schizont stage (Sanz et al., 2013; López-Gutiérrez et al., 2017; Figure 1). Interestingly, UDP-Glc, UDP-GlcNAc and GDP-Man amounts were significantly higher in the sexual stages of the parasite compared to the asexual blood stages (Figure 1), suggesting that certain glycosylations are required for sexual reproduction (López-Gutiérrez et al., 2017).

GDP-Fuc is mostly formed through bioconversion of GDP-Man but is not essential for parasite growth (Sanz et al., 2013; Sanz et al., 2016). In contrast, UDP-GlcNAc is essential because a gene knockout of *Pf*GNA1 (*P. falciparum* glucosamine-phosphate *N*-acetyltransferase), involved in the pathway of UDP-GlcNAc production, causes growth arrest of *Plasmodium* at the late trophozoite stage (Chi et al., 2020). Parasite growth is rescued by supplementing GlcNAc, implying that free GlcNAc can be taken up from external sources (Chi et al., 2020).

Compared to mammals, *P. falciparum* evolved a more promiscuous hexose transporter that imports both glucose and fructose for glycan biosynthesis (Qureshi et al., 2020). This observation is especially interesting considering that glucose uptake is a rate-limiting step during glycolysis, the main energy source for *P. falciparum* (Van Niekerk et al., 2016).

Sugar nucleotide trafficking and hexosamine biosynthesis also play important roles in drug targeting and resistance. For instance, a mutation in the UDP-Gal transporter gene protects *P. falciparum* against imidazolopiperazines, a new class of antimalarial compounds currently examined in clinical trials (Lim et al., 2016). However, this resistance mechanism causes a decrease in parasite fitness as mutants grow more slowly than the wildtype in the absence of the drug (Lim et al., 2016).

On the other hand, inhibition of glucosamine-6-phosphate production in the UDP-GlcNAc synthesis pathway with the drug 6-diazo-5-oxo-L-norleucine (DON) leads to growth arrest at ring or late trophozoite stages, depending on the drug’s concentration. *P. berghei*-infected mice survived longer when treated with DON compared to untreated mice, thus indicating an essential role of the sugar nucleotide for *Plasmodium* viability. Notably, the protection was lost in mice co-injected with GlcN as GlcN is taken up by the parasite directly and converted to GlcNAc, thereby bypassing the DON-inhibited UDP-GlcNAc synthesis pathway (Gomes et al., 2019).

### Glycosylphosphatidylinositols

It was discovered more than 20 years ago that GPI glycolipids are the most abundant glycans on the *P. falciparum* surface, and many GPI-anchored proteins were identified as essential for *P. falciparum* survival (Gerold et al., 1994; Gowda et al., 1997). In addition to their protein-bound form, a major portion of *Plasmodium* GPIs remains devoid of protein (Gerold et al., 1994; Figure 1). These free GPIs contribute to the severity of malaria symptoms and act as proinflammatory toxins, e.g., by inducing TNFα production in macrophages (Schofield and Hackett, 1993). *Plasmodium* GPIs have a conserved core, consisting of a phosphatidylinositol, whose fatty acid chains integrate into the plasma membrane, and an oligosaccharide, composed of one glucosamine (GlcN) followed by three or four mannose (Man) residues. The third mannose carries a phosphoethanolamine (PEtN) moiety that can covalently link the C-terminus of a protein to the GPI.

Database mining recently identified a number of genes involved in biosynthesis of the GPI anchor in *Plasmodium*. The expression of the corresponding enzymes PIG-A, PIG-B, PIG-O, GAA-1, and DPM-1 was validated by immunofluorescence and most proteins were found to localize to the endoplasmic reticulum (ER) (Delorenzi et al., 2002). Another study focused on the addition of the fourth Man to the GPI anchor in *Plasmodium.* Since this step is catalyzed by the enzyme PIG-Z in mammals that is absent in *Plasmodium*, the authors designed PIG-Z-deficient lethal yeast mutants and examined which *Plasmodium* genes can compensate for this knockout (Cortes et al., 2014). In *Plasmodium*, the catalysis task of PIG-Z is undertaken by PIG-B that is responsible for adding only the third Man in mammals, indicating that *Plasmodium* evolved this additional PIG-B functionality to preserve the fourth Man on its GPI.

Further downstream, the GPI transamidase complex can link proteins to the GPI anchor. Notably, mice infected with *P. berghei* carrying a knockout of the *Pb*GPI16 subunit of the GPI transamidase showed less inflammation, and fewer mice challenged with this knockout developed cerebral malaria compared to the wildtype strain (Liu et al., 2018). These findings corroborate the crucial role of *Plasmodium* GPIs in inflammation and the development of severe malaria.

In recent years, the immunogenicity of different parts of the GPI core structure was examined in more detail. Immunizing rabbits with synthetic GPI glycoconjugates yielded polyclonal sera capable of binding to native *Plasmodium* GPIs (Gurale et al., 2016). Vaccinating mice with glycoconjugates composed of synthetic GPI substructures conjugated to a carrier protein revealed the Man_3_-PEtN fragment to be highly immunogenic, while the presence of the fourth Man reduces immunogenicity (Malik et al., 2019). However, only the complete GPI core, also containing GlcN and inositol phosphate (InoP), induced neutralizing antibodies that conveyed protection against *P. berghei* infection. Mice immunized with PEtN-Man_4_-GlcN-InoP showed increased survival, and their TNFα levels stayed low (Malik et al., 2019).

These observations corroborate results obtained from analyzing the naturally elicited antibodies in humans from malaria-endemic regions (Naik et al., 2006; Kamena et al., 2008). Antibodies (Abs) from human serum required the Man_3_-GlcN-InoP substructure as minimal antigen, as binding was lost when shortening the Man chain (Kamena et al., 2008). Similar to mice, the fourth Man did not contribute to antigenicity (Naik et al., 2006). While serum Abs were found to be predominantly directed against the Man core glycan, complete binding was only achieved with the full structure, containing both glycan and lipid moieties (Naik et al., 2006). Comparing serum samples taken at the end of the wet and dry season revealed that Ab titers against *Plasmodium* GPIs were generally increased after the wet season, indicating a correlation with malaria incidence (Kamena et al., 2008).

Since the majority of malaria-caused deaths occur in children below 5 years of age (World Health Organization [WHO], 2020), several studies also examined the anti-GPI Ab titer in serum obtained from young children. Abs targeting *Plasmodium* proteins can be already found in serum from children <12 months, but anti-GPI Abs are only acquired at >18 months of age (Tamborrini et al., 2010). Ab titer correlated with severity of malaria as children with only mild symptoms had Abs even against truncated GPIs but showed less response against the full structure. In contrast, Abs from children with severe symptoms did not recognize truncated GPIs but showed a stronger response against the full structure (Tamborrini et al., 2010). Confirming these observations, high IgG titers against *Plasmodium* GPIs were found in children with enlarged spleens and a recent episode of severe malaria, but low titers in asymptomatic or uninfected children (França et al., 2017).

Abs in the serum of humans from malaria-endemic regions may be used to neutralize GPIs. GPI-induced TNFα production and CD40 upregulation in macrophages were efficiently inhibited by purified GPI-targeting IgGs from individuals with a high anti-GPI Ab titer (De Souza et al., 2010). However, the neutralization capacity of these Abs was reduced when activating macrophages with schizont extract instead of pure GPIs, suggesting that GPIs are not the only proinflammatory toxin present in schizonts (De Souza et al., 2010).

A number of host proteins have been identified as interaction partners of *Plasmodium* GPIs. One major GPI-induced signaling pathway involves Toll-like receptor 2 (TLR2) as GPIs almost completely fail to activate TLR2-deficient macrophages (Krishnegowda et al., 2005). To a lesser extent, GPIs can also induce signaling via TLR4. Supporting evidence comes from competition assays with anti-TLR2 and anti-TLR4 antibodies, that successfully inhibited GPI-induced cytokine secretion (Krishnegowda et al., 2005). In addition, recent molecular docking and dynamics simulations unveiled that both Man and lipid moieties engage with TLR2, thereby inducing and stabilizing the formation of TLR2-TLR1 heterodimers (Durai et al., 2013). To downregulate GPI-induced signaling, the host can degrade free GPIs with phospholipases in the serum or on the macrophage surface (Krishnegowda et al., 2005).

These observations are validated by the finding that also CD36, a TLR2-cooperating receptor, plays an important role in GPI-induced inflammation (Patel et al., 2007). Upon stimulation with GPIs, CD36-deficient macrophages showed reduced phosphorylation of MAPK effectors, reduced TNFα secretion and reduced phagocytosis of *Plasmodium*-infected RBCs compared to the CD36-expressing control cells (Patel et al., 2007). CD36 knockout mice were more susceptible to severe malaria with earlier spikes in parasitemia and increased mortality, highlighting the importance of this signaling pathway for host defense against malaria (Patel et al., 2007). *Plasmodium* GPIs also interact with the host protein moesin *in vitro.* However, moesin-deficient mice were not protected against cerebral malaria and still showed *Plasmodium-*induced cytokine secretion (Dunst et al., 2017).

In the field of vaccine development, a recent study investigated a GPI-conjugate vaccine for malaria transmission blocking. The protein Pfs25, a *Plasmodium* surface antigen and initially promising candidate for a transmission blocking vaccine, lacked potency in clinical trials. To improve its efficacy, Pfs25 was conjugated to a synthetic GPI fragment, comprised of PEtN, the Man_3_-GlcN-glycan core and the inositol moiety without lipids (Kapoor et al., 2018). The PEtN moiety was functionalized with dibenzocyclooctyne, enabling conjugation to Pfs25 via click chemistry. The authors demonstrated that Pfs25-GPI induces higher Ab titers in the serum than unconjugated Pfs25. Abs induced by Pfs25-GPI were able to almost completely abolish *Plasmodium* transmission in membrane-feeding assays with mosquitoes, where Abs raised against unconjugated Pfs25 failed. *Plasmodium* GPIs thus hold great potential for the enhancement of existing protein-based vaccine candidates against malaria (Kapoor et al., 2018).

### *N-*Glycans

*N*-Glycans are linked to proteins via an *N*-glycosidic bond to asparagine. In eukaryotes, *N*-glycosylation starts with a GlcNAc moiety that is extended in a conserved, multi-step pathway in the ER and Golgi to complex, branched carbohydrate structures (Varki et al., 2017).

The presence of *N-*glycans in *Plasmodium* has been under debate for several decades. While evidence from metabolic labeling and enzymatic glycan cleavage experiments indicated the absence of *N-*glycans in *P. falciparum* (Dieckmann-Schuppert et al., 1992), the presence of long *N-*glycans as GlcNAc_2_, Man_3_GlcNAc_2_, and Man_9_GlcNAc_2_ at the ring and trophozoite stage of *Plasmodium* was reported (Kimura et al., 1996). A recent analysis of the *Plasmodium* genome suggested that *Plasmodium* can express most subunits of the oligosaccharyl transferase complex (OST; Tamana and Promponas, 2019), needed to link *N-*glycans to the asparagine of a protein, but evidence for OST expression has not been provided so far. Based on the *Plasmodium* genome, Bushkin et al. (2010) predicted that the parasites are only able to produce short *N-*glycans of two GlcNAc moieties at most (Figure 1). Indeed, metabolic labeling with tritiated GlcN confirmed that GlcNAc and GlcNAc_2_ are produced by *Plasmodium*, and the GlcNAc-recognizing lectin GSL-II showed binding to numerous *P. falciparum* glycoproteins which was lost after *N* glycosidase treatment (Bushkin et al., 2010). GSL-II preferentially localizes to the rhoptry organelle of *Plasmodium* and, to a lesser extent, to the ER and cell surface, but does not recognize the apicoplast, food vacuole or parasitophorous vacuole. These findings indicate that *N-*glycosylation in *P. falciparum* is mostly found on the rhoptry, ER and cell surface, but its respective biological function remains a mystery.

### *O-*Glycans

*O*-Glycans are attached to serine or threonine residues of proteins via an *O*-glycosidic bond. *O*-Glycosylation generally takes place in the Golgi apparatus and begins with a GalNAc moiety. Early glycomics experiments revealed the presence of *O-*glycans in *Plasmodium-*infected RBCs (Dieckmann-Schuppert et al., 1993). Instead of the canonical GalNAc, the reducing end of *Plasmodium O*-glycans is composed of a GlcNAc moiety. GlcNAcylation in *P. falciparum* was confirmed by the discovery of a *Plasmodium O-*GlcNAc transferase (Dieckmann-Schuppert et al., 1993) and by successful purification of *O*-GlcNAcylated proteins from isolated *P. falciparum* parasites (Kupferschmid et al., 2017). At least 13 glycosylated proteins were identified, including Hsp70 and α-tubulin.

### *O-*Fucosylation

**FIGURE 2:**
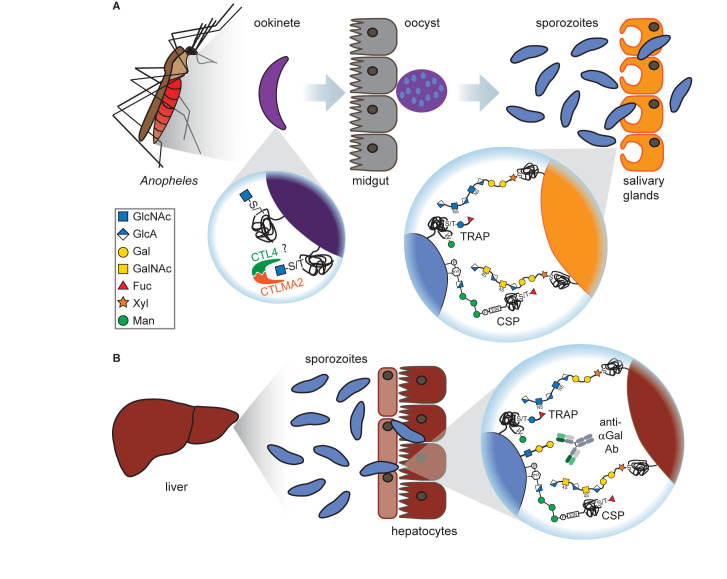
Glycans at the mosquito and liver stages of *Plasmodium.* **(A)** Ookinetes carry short *O-*GlcNAcylations on glycoproteins on their surface which presumably bind to the C-type lectins CTL4 and CTLMA2, thereby preventing melanization through the mosquito’s immune system. After oocyst rupture at the mosquito midgut, released sporozoites express the glycoproteins TRAP and CSP. Both proteins are *O-*fucosylated and can interact with GAGs on the surface of *Anopheles* salivary gland cells, although this interaction is dispensable for salivary gland penetration. **(B)** Upon arrival at the liver, CSP and TRAP bind to the GAGs on hepatocytes which is a prerequisite for hepatocyte invasion. Sporozoites have been found to express the αGal antigen that elicits a high Ab titer in humans. This is particularly interesting as vaccination of mice with αGal conveyed protection against malaria transmission.

A proteome-wide mass spectrometry screen of sporozoite surface proteins revealed that *Plasmodium* is also capable of *O-*fucosylation (Lopaticki et al., 2017), as this modification was identified on the two *Plasmodium* proteins circumsporozoite protein (CSP) and thrombospondin-related anonymous protein (TRAP; see Figure 2). *O*-Fucosylation is a non-canonical modification carried out by *O*-fucosyltransferases at special consensus sequences, including the thrombospondin type 1 repeats found in CSP and TRAP (Holdener and Haltiwanger, 2019).

While CSP appeared both with and without fucosylation, TRAP was only present in glycosylated form, modified either with a single fucose or a glucosylfucose dimer. These observations were validated by the discovery of an *O*-fucosyltransferase in *P. falciparum* (POFUT2), that is responsible for CSP and TRAP glycosylations and essential for mosquito midgut colonization of *P. falciparum* sporozoites (Lopaticki et al., 2017).

### *C-*Mannosylation

Interestingly, TRAP was also found to be *C*-mannosylated (Swearingen et al., 2016; Hoppe et al., 2018), a rare glycosylation form where a mannose moiety is attached to the indole ring of a tryptophan (Figure 2). This modification is catalyzed in *Plasmodium* by the *C*-mannosyltransferase DPY19 but is not essential for parasite survival as depletion of DPY19 does not impair proliferation and development during the asexual blood stages (Hoppe et al., 2018; López-Gutiérrez et al., 2019). Since both proteins are important vaccine targets, it has been hypothesized that their glycosylations may impact the antigenicity of CSP and TRAP (Swearingen et al., 2016).

### α-Gal Antigen

The trisaccharide α-gal (Galα1–3Galβ1–4GlcNAc-R, also known as Galili antigen) was found on *Plasmodium* sporozoites (Yilmaz et al., 2014; Figure 2). While humans have lost the ability to synthesize this glycan due to inactivation of the *α1,3GT* gene, many organisms express this glycan, including bacteria found in the human gut microbiota. Therefore, human serum contains large quantities of naturally occurring α-gal Abs. Interestingly, the presence of α-gal Abs in humans from malaria-endemic regions correlated with a lower risk for *Plasmodium* infection (Yilmaz et al., 2014). Anti-*α*-gal Abs activate complement-dependent cytotoxicity against *Plasmodium* sporozoites and inhibit hepatocyte invasion *in vitro.* Vaccination of *α1,3GT*-depleted mice with *α-*gal conveyed protection against malaria transmission *in vivo* (Yilmaz et al., 2014). *α-*Gal surface expression is lost on the later, asexual blood stages of *Plasmodium* (Ramasamy and Field, 2012), rendering *α-*gal an attractive candidate for a transmission blocking vaccine, possibly in combination with other *Plasmodium* antigens.

## Host Glycans

### ABO Antigens

The ABO antigens are carbohydrate portions of glycoproteins and glycolipids that terminate with different monosaccharides. Terminal *N*-acetylgalactosamine or galactose determine the A or B blood group, respectively. These sugars cap the core structure Fuc(α1–2)Gal(β1–3)GlcNAc(β1–3)Gal called the H antigen (Table 1). The A and B antigens are produced by human ABO transferase, which transfers GalNac or Gal onto the core structure, depending on missense single nucleotide polymorphisms in the ABO gene. Inheriting two null copies of the ABO gene results in the O blood group (the H antigen remains uncapped). Glycoproteins and glycolipids carrying the ABO antigens are present on the surface of RBCs, endothelial, and epithelial cells (only in secretors) (Yamamoto et al., 1990).

Decades ago, epidemiological data from malaria-endemic regions suggested that the severity of malaria depends to some extent on the blood group [reviewed in Cserti and Dzik (2007) and Uneke (2007)]. However, different serological studies produced ambiguous results, requiring a more precise examination method. Genotyping almost 10,000 individuals found that individuals with blood groups A, B, and AB are more susceptible to severe malaria than blood group O (Fry et al., 2008), validating previous conclusions (Cserti and Dzik, 2007; Uneke, 2007).

**FIGURE 3:**
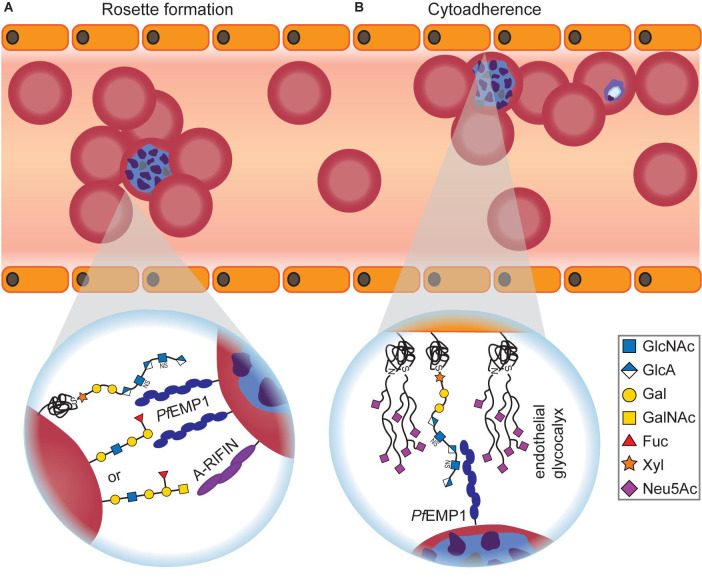
Glycan-dependent interactions between *Plasmodium-*infected RBCs and uninfected host cells. **(A)** Infected RBCs form rosettes with uninfected RBCs, thereby becoming invisible for the host’s immune system. Depending on the blood group, *Plasmodium* uses different surface proteins for rosetting. *Pf*EMP1 recognizes O-type RBCs, while A-RIFIN binds to A antigen. Independent of the blood group, *Pf*EMP1 also interacts with RBC GAGs for rosette formation that can be efficiently inhibited by adding soluble GAGs *in vitro* and *in vivo.* **(B)** Infected RBCs adhere to endothelial cells to avoid blood clearance. The interaction between *Pf*EMP1 and GAGs, predominantly heparan sulfate, is crucial for cytoadherence of infected RBCs. On the other hand, it was shown that an intact endothelial glycocalyx conveys protection against *Plasmodium* cytoadherence as severe malaria symptoms are preceded by degradation of the endothelial glycocalyx.

The molecular basis for blood-group-dependent differences was investigated as well (Figure 3A). Rosetting, the ability of *P. falciparum*-infected RBCs to build clusters with uninfected RBCs, is one determinant for severe malaria and has been suggested to involve blood group antigens (Barragan et al., 2000). When examining rosette formation of multiple clinical isolates of *P. falciparum*, the majority of isolates preferred blood group A or B, but none preferred blood group O (Barragan et al., 2000). Rosette formation in the preferred blood group was inhibited by addition of the respective soluble blood group antigen, and the blood group preference was lost after treatment of RBCs with glycosidases (Barragan et al., 2000). Blood group antigens A and B thus appear to be important co-receptors for rosette formation of infected RBCs.

ABO-decorated giant unilamellar vesicles (GUVs) were used as RBC mimetics to examine rosette formation (Vagianou et al., 2018). A- and O-type GUVs participated in rosettes, but not B-type GUVs, and rosetting of O-type GUVs was inhibited with anti-*Pf*EMP1 Abs. Therefore, the different blood groups involve at least two distinct pathways for rosette formation: *Pf*EMP1-dependent for O-type RBCs or *Pf*EMP1-independent for A-type RBCs, respectively (Vagianou et al., 2018; Figure 3A). The *Pf*EMP1-independent rosetting pathway involves the *Plasmodium* protein A-RIFIN (repetitive interspersed families of polypeptides) instead (Goel et al., 2015). Indeed, CHO cells that overexpress A-RIFIN from *Plasmodium* sequester A-type RBCs but do not bind to B- or O-type RBCs (Goel et al., 2015).

*In vitro* invasion experiments with blood from the rare Bombay type O^h^ that lacks the H antigen of blood group O revealed that O^h^-type RBCs, i.e., without any blood group antigens, are more resistant to *Plasmodium* than blood group O (Pathak et al., 2016). This resistance was emulated in O-type RBCs by the addition of lectins against H antigen, suggesting that the presence of a blood group antigen is essential for *P. falciparum* invasion (Pathak et al., 2016).

### Glycosaminoglycans

Glycosaminoglycans (GAGs) are extracellular matrix components of mammalian cells, consisting of acidic disaccharide repeating units which show varying degress of sulfation. GAGs have many crucial functions in humans, including cell–cell adhesion and tissue integrity but also tissue hydration and elasticity (Varki et al., 2017).

During *Plasmodium* infection, CSP recognizes the GAGs heparan sulfate and chondroitin sulfate on the surface of human hepatocytes and RBCs (Pancake et al., 1992; Frevert et al., 1993; Rogerson et al., 1995; Figures 1, 2B). The addition of soluble GAGs *in vitro* and *in vivo* can inhibit this interaction and thereby prevent sporozoite invasion in human cells, raising hopes for the therapeutic exploitation of GAGs (Pancake et al., 1992). Interestingly, heparan sulfate and chondroitin sulfate are also present in the salivary glands of *Anopheles gambiae* but seem dispensable for sporozoite invasion in the mosquito (Figure 2A). Silencing of the GAG synthesis enzyme *Ag*OXT1 only marginally reduced sporozoite invasion, indicating that additional interactions contribute to *Plasmodium* invasion in the mosquito midgut (Armistead et al., 2011).

At the blood stages of the *Plasmodium* life cycle, GAGs have been implicated in rosette formation (Figure 3A) and adherence of infected RBCs to the human placenta during pregnancy, as soluble GAGs disrupt both processes (Fried and Duffy, 1996; Barragan et al., 1999; Chotivanich et al., 2012). The inhibitory effect of GAGs on rosetting strongly depends on the sulfation state of the glycans (Barragan et al., 1999). Adherence of infected RBCs to placental cells involves the unsulfated GAG hyaluronic acid (HA; Beeson et al., 2000). Since CD44 on RBCs is a known HA receptor, whose recognition of HA can be manipulated by differential glycosylation (Katoh et al., 1995), it is conceivable that CD44 might also be involved in placental adherence of infected RBCs. Furthermore, CD44 mediates adherence of infected RBCs to brain endothelial cells in cerebral malaria via the GAG chondroitin sulfate (Jurzynski et al., 2007). Notably, a knockout of CD44 in RBCs inhibited *Plasmodium* invasion as well, suggesting a broader role of CD44 in the life cycle of the parasite (Kanjee et al., 2017). In addition to GAGs, adherence to placental cells might also involve interaction with Lewis glycan antigens, further complicating the matter (Hromatka et al., 2013).

In order to gain insights on the localization and identity of heparin-binding *Plasmodium* proteins, the heparin interactome has been examined by mass spectrometry and immunoblots (Kobayashi et al., 2013; Zhang et al., 2013, 2014). All identified proteins were part of the Duffy and reticulocyte binding-like families (Kobayashi et al., 2013). Staining of merozoites with biotinylated heparin for confocal microscopy revealed that heparin localized mostly to the apical tip and the rhoptry organelle of the parasite which causes invasion inhibition (Kobayashi et al., 2013; Zhang et al., 2013). Heparin also inhibits merozoite egress during schizogony, by blocking *Plasmodium-*induced RBC membrane pores through simultaneous binding to the inner RBC membrane and the merozoite surface (Glushakova et al., 2017).

Two *Plasmodium* proteins highlight the crucial importance of GAG interactions for the parasite: *Pf*EMP1, found on infected RBCs, plays an essential role in GAG-dependent cytoadherence (Vogt et al., 2003; Figure 3B) and rosette formation (Vagianou et al., 2018; also see previous section) as its DBL1α domain can recognize heparan sulfate on the surface of epithelial cells and anti-*Pf*EMP1 Abs disrupt rosetting. Heparan sulfate on the RBC surface is recognized by the protein *Pf*BAEBL that also binds sialic acids on RBCs (see next section), suggesting an alternative recognition pathway independent of sialic acid (Kobayashi et al., 2010).

Considering that heparin is a widely used drug, the inhibitory effect of soluble GAGs against *Plasmodium* invasion and cytoadherence raised hopes for a potential therapeutic exploitation. However, the strong anticoagulating activity of heparin poses a risk for possible antimalarial use because it can cause undesired bleeding. Recent studies examined heparin-like alternatives, in search for compounds without anticoagulating activity but an inhibitory effect on *Plasmodium* invasion. Heparin-like polysaccharides isolated from the capsule of the *E. coli* K5 strain were tested for anti-invasive effects (Boyle et al., 2010). The compound repertoire was expanded by chemical modification of the K5 polysaccharides and heparin, and the key properties of a suitable drug were identified (Boyle et al., 2017). For optimal antimalarial activity, heparin-like molecules (HLMs) should contain at least six monosaccharides and two sulfations per repeating unit with disulfation of a HexA or GlcNAc moiety (Boyle et al., 2010, 2017). Chemical modification (e.g., hypersulfation) can improve invasion inhibition and lower the anticoagulating effect (Boyle et al., 2017).

Based on these results, semi-synthetic non-GAG HLMs with antimalarial activity but little anticoagulating activity were successfully developed (Skidmore et al., 2017). The antimalarial activity of such non-GAG HLMs apparently depends on sulfation as the non-GAG HLMs showing the highest inhibition of *Plasmodium* cytoadherence, glycogen type 2 sulfate and phenoxyacetylcellulose sulfate, are both heavily sulfated (Skidmore et al., 2017).

Heparin-like molecules also proved beneficial against blood–brain barrier (BBB) breakdown in an *in vitro* model of cerebral malaria (Moxon et al., 2020). Accumulation of parasite and RBC histones at the brain endothelium, likely released during schizont rupture, leads to BBB disruption. HLMs prevent histone-induced BBB breakdown, suggesting yet another pathway for therapeutic application of HLMs (Moxon et al., 2020).

Naturally derived HLMs from marine organisms were examined for their antimalarial efficacy. Heparan sulfate isolated from lion’s paw scallops and fucosylated chondroitin sulfate isolated from sea cucumbers efficiently inhibit merozoite invasion and cytoadherence *in vitro* (Bastos et al., 2014, 2019). Both GAGs can be readily obtained from natural sources and showed less anticoagulating activity than heparin. Additional HLMs were purified from other types of sea cucumber, red algae and marine sponges, with the majority of marine HLMs exhibiting antimalarial activity on a similar level as heparin but with less anticoagulating side effects (Marques et al., 2016). HLM injection also conveyed protection against *P. yoelii in vivo* as infected mice showed increased survival and decreased parasitaemia in the serum when injected with marine HLMs. Interestingly, surviving, HLM-treated mice displayed high Ab titers against *P. yoelii* antigens and survived re-challenge with the parasite after several months without any HLM treatment. It is thus conceivable that because HLMs disrupt merozoite invasion, the parasites become more exposed to the immune system (Marques et al., 2016).

### Sialic-Acid-Containing Glycans

Sialic acids are a group of sugars that commonly cap the termini of *N-* and *O-*glycan chains. The predominant sialic acids in mammals are *N*-acetylneuraminic acid (Neu5Ac) and *N*-glycolylneuraminic acid (Neu5Gc). While most mammals synthesize Neu5Gc from Neu5Ac, humans lack the respective hydroxylase due to a mutation in their *CMAH* gene and thus do not possess sialic acid in the Neu5Gc form (Chou et al., 1998).

Starting from the mid-1980s, evidence for an interaction between the *P. falciparum* erythrocyte-binding antigen 175 (EBA-175) and sialic acid on the host protein glycophorin A (Camus and Hadley, 1985; Orlandi et al., 1992; Sim et al., 1994) was unearthed. This interaction is crucial for RBC invasion in the so-called sialic-acid-dependent pathway (Figure 1) and was validated by structural data, showing that Neu5Ac(α2–3)Gal is making essential contacts to EBA-175 (Tolia et al., 2005). Data derived from glycosylation mutants of glycophorin A revealed that an *O-*glycosylation motif encoded by exon 3 is critical for invasion via the sialic-acid-dependent pathway (Salinas et al., 2014).

The interaction between the polymorphic EBA-140 and glycophorin C also contributes to invasion via the sialic-acid-dependent pathway (Lobo et al., 2003), and a structure of EBA-140 in complex with sialic acid confirms this interaction (Malpede et al., 2013). An *N-*glycan on glycophorin C that was subsequently identified, is recognized by one variant of EBA-140, suggesting that not only *O-*glycans are involved in the sialic-acid-dependent pathway of invasion (Mayer et al., 2006). When both glycophorin A and C are enzymatically cleaved, *P. falciparum* binds to glycophorin B for invasion (Dolan et al., 1994). Although glycophorin B shows a high degree of sequence similarity to glycophorin A, it is not recognized by EBA-175 but by the *Plasmodium* protein EBL-1 instead (Li et al., 2012).

Some strains of *P. falciparum* are capable of invading RBCs after enzymatic cleavage of sialic acid with neuraminidase, proving the existence of an alternative, sialic-acid-independent pathway (Hadley et al., 1987). Even strains that usually depend on sialic acid for invasion can switch to the independent pathway when EBA-175 is depleted (Duraisingh et al., 2003).

Both pathways can be efficiently targeted by Abs against EBA-175 and RH5, a protein involved in the sialic-acid-independent pathway (Rodriguez et al., 2008). Ord et al. (2012) raised Abs in mice against both proteins and found that these Abs can block invasion. However, only the anti-RH5 Ab inhibited invasion of neuraminidase-treated RBCs, demonstrating that the sialic-acid-independent pathway needs to be disabled as well to hamper invasion. Recent evidence suggests another protein to participate in the sialic-acid-dependent pathway: *P. falciparum* rhoptry associated adhesin (*Pf*RA), that is only expressed at the schizont stage and localizes to the apical merozoite surface during RBC invasion (Anand et al., 2016). *Pf*RA fails to bind neuraminidase-treated RBCs, and RBC invasion can be inhibited by anti-*Pf*RA Abs (Anand et al., 2016).

*Plasmodium knowlesi* can transmit from Neu5Gc-producing macaques to humans, raising questions about the sialic-acid-dependent invasion pathway of this *Plasmodium* species (Dankwa et al., 2016). When reconstituting the CMAH enzyme in human RBCs, CMAH+ RBCs become more susceptible to *P. knowlesi* invasion, and Neu5Gc-containing receptors are specifically recognized by the *P. knowlesi* proteins DBPβ and γ (Dankwa et al., 2016). A human-adapted strain of *P. knowlesi* did not require Neu5Gc and lost DBPγ expression, suggesting a shift toward sialic-acid-independent invasion due to selection pressure (Dankwa et al., 2016). Other *Plasmodium* species show a more exclusive preference for one of the two sialoforms. While *P. reichenowi* and *P. falciparum* are genetically very similar, they only infect chimpanzees or humans, respectively (Martin et al., 2005). This difference in host specificity is attributed to preferential binding of *Pf*EBA-175 to Neu5Ac and *Pr*EBA-175 to Neu5Gc. Furthermore, *P. reichenowi* and other ape-specific *Plasmodium* species express EBA-165 that also preferentially recognizes Neu5Gc (Proto et al., 2019). In contrast, *P. falciparum* does not express EBA-165 due to a frame-shift mutation and actively silenced EBA-165 when the frame-shift was corrected by CRISPR-Cas9 editing (Proto et al., 2019). The identity of the recognized sialoprotein also differs between *P. reichenowi* and *P. falciparum.* While *Pf*EBA-140 binds to glycophorin C as described above, the *P. reichenowi* homolog interacts with glycophorin D (Zerka et al., 2017). These observations favor a model in which humans and *P. falciparum* co-evolved and *P. falciparum’s* ability to preferentially recognize Neu5Ac-containing proteins led to its emergence as the most deadly human parasite (Martin et al., 2005; Proto et al., 2019).

Sialylation of *N-* and *O-*glycans as well as sulfation of GAGs are both critical for *Plasmodium* cytoadherence and invasion. Both glycan modifications introduce charge to the carbohydrate structure. Thus, it seems likely that ionic interactions are required for the interplay between host and parasite when these carbohydrate moieties are involved. Detailed structural data will be needed to examine this hypothesis.

### *N-*Glycans

Sickle-cell disease conveys resistance against severe malaria. However, *Plasmodium* infects healthy and sickle-cell RBCs equally well with no apparent differences in invasion or release (Friedman, 1978), suggesting that resistance arises from more efficient immune clearance of infected RBCs. RBCs with sickle-cell trait were recently found to express high-mannose *N-*glycans on their surface that are recognized by the macrophage receptor CD206 followed by phagocytosis (Cao et al., 2021). High-mannose *N-*glycan surface levels in sickle-cell RBCs correlated with the parasite’s life stage, being elevated at trophozoite and schizont stage (see Table 1). Phagocytosis through macrophages can be inhibited by the addition of mannan, a yeast-derived high-mannose glycan, and oxidative stress can induce expression of high-mannose surface *N-*glycans in healthy RBCs (Cao et al., 2021). Improved immune clearance presumably arises from the increased susceptibility of sickle-cell RBCs to oxidative stress caused by the parasite, mediated through an elevated high-mannose *N-glycan* level recognized by macrophages (Cao et al., 2021).

*Plasmodium* also alters the *N-*glycosylation pattern of infected RBCs without sickle-cell trait. Extensive posttranslational modification-omics screens with infected RBCs at the different asexual life stages of *Plasmodium* (Wang et al., 2021) revealed that *Plasmodium* downregulates *N-*glycosylation of a variety of RBC proteins (Figure 1), including several cluster of differentiation (CD) proteins with known roles in cell adhesion and rosette formation. For the majority of RBC membrane proteins, *N-*glycosylation decreased over the course of the *Plasmodium* life cycle with the lowest glycosylation level at the late schizont stage (Wang et al., 2021). For instance, *N*-glycosylation of the C3 complement protein recedes, suggesting a parasitic strategy to dampen the host immune response. In contrast, *N*-glycosylation of selected RBC proteins is upregulated during parasite development inside the RBC (Wang et al., 2021). Notably, CD55 displays higher levels of *N-*glycosylation which is especially interesting because it has been shown to be an essential receptor in *Plasmodium* invasion (Egan et al., 2015). The exact mechanism behind the observed global changes in RBC glycosylation or their biological purpose is still a mystery. However, the simultaneous increase in *Plasmodium* sugar nucleotide levels (Sanz et al., 2013; López-Gutiérrez et al., 2017; see above) suggests that *Plasmodium* utilizes host glycans for purposes yet to be discovered.

## Biological Functions

### Vector Colonization

First evidence for a crucial role of *Plasmodium* glycans in mosquito midgut invasion came from the observation that ookinetes lose their ability to invade the midgut upon treatment with the GlcNAc-binding lectin wheat germ agglutinin, suggesting that carbohydrate binding is required at this step (Basseri et al., 2016). Since the lectin concanavalin A that predominantly binds mannose structures did not inhibit invasion, the involved carbohydrate likely contains GlcNAc or sialic acid but not mannose or glucose (Figure 2A). This notion is confirmed by the finding that a number of *O-*GlcNAcylated proteins have been identified in *Plasmodium* (Kupferschmid et al., 2017). In addition, the *O-*fucosylation on the sporozoite proteins CSP and TRAP is essential for midgut colonization (Lopaticki et al., 2017).

Interestingly, it has been described that after infection with *P. falciparum*, the probing activity of *Anopheles* mosquitoes increases, coinciding with a higher sugar uptake of the mosquito at the *Plasmodium* oocyst stage. Sugar uptake decreases subsequently when parasites reach the sporozoite stage. This argues in favor of a model in which the parasite controls and manipulates the behavior of its vector to increase its sugar supply (Nyasembe et al., 2014).

Investigating potential vector-sided interaction partners of *Plasmodium* glycans, two C-type lectins in *Anopheles* were identified, CTL4 and CTLMA2 (Osta et al., 2004; Figure 2A). Both lectins can form heterodimers for a cooperative binding mode and recognize specific glycosaminoglycan motifs, including β1–3- and β1–4-connected Glc, Gal, GlcNAc and GalNAc moieties (Bishnoi et al., 2019). Depletion of the two proteins in *Anopheles* enables a strong immune response against *Plasmodium* followed by melanization, an innate defense mechanism of *Anopheles* leading to sequestration and melanin coating of the parasite. Therefore, parasite binding to these lectins conveys protection against the mosquito’s immune response (Osta et al., 2004).

### Host Cell Invasion and Immune Defense

We have already touched on various glycans involved in *Plasmodium* invasion above. At the host cell surface, the sialic-acid-dependent pathway as well as the crucial roles of GAGs and blood group antigens were discussed in detail.

These glycans are part of the host cell glycocalyx that was found to be one of the main determinants of malaria resilience. For instance, loss of the glycocalyx on brain endothelial cells is one event preceding cerebral malaria. Transmission electron microscopy revealed that the brain endothelial glycocalyx of mice challenged with uncomplicated *P. chabaudi* was only partially disrupted, whereas it was completely degraded in mice with *P. berghei*-induced cerebral malaria (Hempel et al., 2014). Glycocalyx degradation coincided with an increase in circulating GAGs and was already present before other symptoms of cerebral malaria occurred (Hempel et al., 2014). In a later study, the same group investigated how an intact glycocalyx can convey protection against severe malaria symptoms. The ability of *Plasmodium* to bind to the surface receptor CD36 was examined on CHO cells which develop a thick glycocalyx within 4 days *in vitro* (Hempel et al., 2017). *Plasmodium* binding to CD36 was lost with the maturation of the glycocalyx over time, suggesting that the glycocalyx serves as a shield that can prevent *Plasmodium* cytoadhesion to the endothelium (Hempel et al., 2017; Figure 3B). These findings are corroborated by microfluidics experiments examining the interaction between infected RBCs and the glycocalyx (Introini et al., 2018). After artificial disruption of the glycocalyx with sialidases, cytoadhesion of infected RBCs is significantly increased (Introini et al., 2018; Figure 3B). Disruption of the glycocalyx caused by *Plasmodium* in cerebral malaria can be prevented *in vivo:* when treating infected mice with corticosteroids or antithrombin-3, the glycocalyx stays intact, and cerebral malaria symptoms as well as mortality are markedly reduced, suggesting glycocalyx integrity as a promising leverage point for enhanced host resilience (Hempel et al., 2019).

Depletion of GlcNAc-transferase V, which adds β1–6-connected GlcNAc to *N-*glycans, renders mice more susceptible to malaria, as marked by higher parasitemia, loss in body weight and more severe pathology in the liver and lung (Shibui et al., 2011). GlcNAc-containing *N-*glycans may convey some degree of protection against severe malaria, but the mechanistic details of this putative protection remain unclear (Shibui et al., 2011).

Lectins are another group of important modulators in the host defense against *Plasmodium*. Mannose-binding lectin (MBL) that triggers the lectin pathway of the complement system is crucial for the host’s defense against malaria. It is especially important for resilience to placental malaria as the adaptive immune system has not yet formed in the fetus. Recently, a correlation between susceptibility to placental and non-placental malaria and single nucleotide polymorphisms in the *MBL* gene was established (Holmberg et al., 2012; Jha et al., 2014). Some variants caused an increased risk for severe malaria while others conferred protection. Counterintuitively, variants with decreased susceptibility to placental malaria dampened complement activation. However, it is conceivable that the protective effect of a reduced complement response lies in the prevention of hyperinflammation at the placenta (Holmberg et al., 2012).

Galectins constitute another lectin type that specifically recognizes β-galactosides as part of *N-* and *O-*glycans. Interestingly, host galectins also seem to be involved in controlling *Plasmodium* infection. Inhibition of galectins through lactose injection led to increased mortality of *Plasmodium*-infected mice (Liu et al., 2016). Lactose-treated mice exhibited increased parasitaemia, lung pathology and expression of interferon α/β/γ and interleukines 4/10 in the lung (Liu et al., 2016). These data suggest that galectins can protect from *Plasmodium* infection. This hypothesis is supported by the observation that galectin-9 expression was significantly upregulated on macrophages in the lungs of infected mice (Liu et al., 2016). In contrast, galectin-3 apparently increases the susceptibility to infection with some *Plasmodium* species as galectin-3-deficient mice show reduced parasitaemia compared to wildtype mice when challenged with *P. yoelii or P. chabaudi* but not *P. berghei.* The galectin-3 knockout mice infected with *P. yoelii* were also able to raise higher Ab titers against *Plasmodium* antigens than the wildtype, suggesting that galectin-3 modulates the immune response against *Plasmodium* in a non-beneficial way (Toscano et al., 2012). The precise role of galectins in malaria host defense remains to be elucidated, but it seems likely that their modulatory effect on the immune response is *Plasmodium* species-dependent and involves additional factors.

In some cases, *Plasmodium* proteins can also prevent immunomodulatory host glycan–protein interactions. It has been reported that *Plasmodium* merozoite surface protein 7 (MSP7) binds to the host’s C-type lectin P-selectin (Perrin et al., 2015). P-selectin plays a crucial role in leukocyte recruitment to inflamed endothelial tissue by binding to the glycan sialyl-Lewis X on leukocytes. Binding of MSP7 inhibits the interaction between P-selectin and sialyl-Lewis X (Perrin et al., 2015). It seems conceivable that the proinflammatory, immune-system-recruiting function of P-selectin is abrogated by MSP7, indicating a novel pathway for host immune system attenuation by *Plasmodium.*

## Discussion and Outlook

One of the major challenges in the global fight against malaria is the paucity of an effective malaria vaccine, with the only EMA-approved vaccine RTS,S lacking efficacy in the most vulnerable groups of infants and young children (Agnandji et al., 2014). Recently, a new vaccine candidate named R21 was examined in a phase III clinical trial and found to be more effective than RTS,S with approx. 77% efficacy (Datoo et al., 2021). Since both RTS,S and R21 are based on the glycoprotein CSP, a potential effect of CSP glycosylation on vaccine efficacy should be examined. CSP is *O-*fucosylated in *P. falciparum* but not in yeast where CSP for the vaccines is recombinantly expressed (Agnandji et al., 2014; Swearingen et al., 2016; Lopaticki et al., 2017; Datoo et al., 2021). Therefore, alternative expression systems might be considered for malaria vaccine design to obtain bona fide *Plasmodium* glycosylation.

The *Plasmodium* GPI glycan itself is another promising vaccine candidate, as it is highly immunogenic and a critical proinflammatory toxin contributing to severe malaria. GPI vaccine candidates have been examined, but the feasibility and efficacy of a potential GPI vaccine remains controversial due to inconsistent data, most probably caused by using different glycans, immunization regimes and infection models. Detailed immunogenicity studies in mice determined that a minimal antigen for vaccination should at least comprise the mannose glycan core and the phosphoethanolamine moiety of the GPI structure (Malik et al., 2019; see also GPI section). The combination of protein antigens and GPIs proved particularly effective as transmission-blocking vaccine in mice (Kapoor et al., 2018), suggesting the use of *Plasmodium* GPIs as vaccine adjuvants.

Glycosaminoglycans represent yet another attractive pharmaceutical target. With heparin as an approved and widely used anticoagulant, one prominent GAG is already marketed. The finding that heparin and HLMs can prevent *Plasmodium* invasion renders GAGs attractive antimalarial compounds, and HLMs can avoid the anticoagulating activity of heparin that is undesired for malaria treatment. Indeed, the HLM Sevuparin was found to be non-toxic and well-tolerated in phase I/II human clinical trials (Leitgeb et al., 2017). GAG interactions are also the focus of transmission-blocking vaccines. For instance, *Plasmodium* proteins bind to placental chondroitin sulfate A for invasion, causing placental malaria in pregnant women. Abs inhibiting *Plasmodium* binding to placental GAGs were successfully induced in a phase I clinical trial with the PAMVAC vaccine candidate (Mordmüller et al., 2019).

Another challenge arises from the variability of the *Plasmodium* ssp. While some lectins and glycans, such as GPIs, are conserved across different *Plasmodium* species, it seems likely that their respective interaction partners at the host level vary between distinct host species. For instance, *P. knowlesi* shifts to the sialic acid-independent pathway when infecting humans instead of macaques due to the absence of Neu5Gc on human cells (Dankwa et al., 2016). It is therefore crucial to examine the species-specific differences in *Plasmodium-*host interplay in more detail.

Recent work revealed that *Plasmodium* parasites employ extracellular vesicles (EVs) for cell–cell communication and host cell modulation. Infected RBCs release EVs with diverse cargo, ranging from functional miRNA that changes the barrier properties of host endothelial cells (Mantel et al., 2016) to DNA that promotes sexual differentiation of neighboring parasites (Regev-Rudzki et al., 2013). EVs can also carry 20S proteasomes that change the cytoskeletal architecture of naïve RBCs, thereby priming them for subsequent *Plasmodium* infection (Dekel et al., 2021). While nucleic acid and protein cargo of *Plasmodium-*derived EVs are intensively studied, the presence, role or origin of glycans carried on the cell membrane of secreted EVs remains to be elucidated.

While recent work has provided important insights about *Plasmodium* glycans, many open questions remain, mostly due to the lack of tools targeting specific glycans. Monoclonal anti-glycan antibodies are scarce and lectins, though often used to examine the terminal moieties of glycans, are not specific enough. Novel glycan-targeting probes will allow for a more in-depth characterization of the *Plasmodium* glycome and facilitate therapeutic applications against drug-resistant parasites.

## Author Contributions

FG and OM designed and wrote the review. PS revised the manuscript. All authors contributed to the article and approved the submitted version.

## Conflict of Interest

The authors declare that the research was conducted in the absence of any commercial or financial relationships that could be construed as a potential conflict of interest.
